# Comparative Assessment on Selected Physicochemical Parameters and Antioxidant and Antimicrobial Activities of Honey Samples from Selected Districts of the Amhara and Tigray Regions, Ethiopia

**DOI:** 10.1155/2019/4101695

**Published:** 2019-03-04

**Authors:** Mekuanint Lewoyehu, Meareg Amare

**Affiliations:** ^1^Department of Natural Resource Management, College of Agriculture and Environmental Sciences, Bahir Dar University, P.O. Box,79, Bahir Dar, Ethiopia; ^2^Department of Chemistry, College of Science, Bahir Dar University, P.O. Box 79, Bahir Dar, Ethiopia

## Abstract

The composition and properties of natural honeys differ with plant species on which the bees forage and the climatic conditions of the production areas. In Ethiopia, Amhara and Tigray are neighboring regions consisting of different agricultural activities and blossoms from different types of vegetations which may influence the natural composition and hence the properties of honey. So, the aim of the current study was to assess the quality of honey from selected districts of the two regions. In the study, 18 composited honey samples were collected from six selected districts and analyzed for selected physicochemical parameters and antioxidant and antimicrobial activities. The analyses of physicochemical parameters were carried out following standard procedures of IHC and QSAE. The antioxidant activity was determined by analyzing the RSA using DPPH while the antibacterial activities were determined by the agar well diffusion method. The moisture, ash content, electrical conductivity, pH, free acidity, reducing sugar, and sucrose content of the honey samples were found to be in the range 16.34 ± 0.26 to 19.83 ± 0.43 %, 0.08 ± 0.00 to 0.45 ± 0.03 %, 0.19 ± 0.00 to 0.89 ± 0.03 mS/cm, 3.79 ± 0.04 to 4.20 ± 0.01, 19.56 ± 1.13 to 38.11 ± 1.54 meq/kg, 62.10 ± 0.48 to 66.37 ± 0.20 %, and 1.35 ± 0.08 to 5.96 ± 0.10 %, respectively. The total phenolic content ranged from 1165.60 ± 23.45 to 1854.83 ± 10.47 mg/kg with antioxidant activity of 21.64 ± 0.26 to 36.12 ± 0.52 AEAC/100 g. The total phenolic contents showed strong correlation with RSA. Furthermore, all honey samples showed an antibacterial activity varying from 23.23 ± 0.12 to 28.84 ± 0.24 mm.

## 1. Introduction

Honey is the natural sweet substance produced by honey bees from the nectar or secretions of living parts of plants [1]. It is a complex mixture with very great variations in composition and characteristics due to its geographical and botanical origin, the floral origin, or the nectar utilized by bees [2].

Research results indicated functional properties of honey in human health promotion which depend largely on the floral sources of the honey, its high osmolarity, and antibacterial properties [3] triggering its wound dressing properties which might be ascribed to its antioxidant and antibacterial activities.

While Ethiopia is the first in Africa and third in the world in beeswax production [4], it is the leader in Africa and 10^th^ in the world in honey production. The high honey production potential of Ethiopia is attributed to its tremendous varieties of agroclimatic conditions and biodiversity which favored the existence of diversified honey bee flora and huge number of honeybee colonies [5]. Ethiopia is reported to have over 10 million bee colonies of which 5 to 7.5 million are hived while the remaining are wild [6]. According to CSA and Agricultural sample survey [7, 8], the major honey and beeswax producing regions in Ethiopia are Oromia (41%), Amhara (22%), SNNPR (21%), and Tigray (5%).

The quality of honey being a key factor for both local and international markets [9] is an aspect disregarded by producers and processors in developing countries including Ethiopia. Due to this, thousands of tons of honey produced every year are usually crude, poorly managed, and unattractive in appearance [10].

The composition and quality of honey in general are greatly influenced by geographical and environmental factors and the types of flowers utilized by bees [11]. Since Amhara and Tigray are the neighboring regions in Ethiopia known to produce white honey, the honey produced in Tigray is prestigiously white, presumed by Ethiopian users to have a medicinal value, and hence is more expensive than honey produced in Amhara [8, 12].

To the best of our knowledge, although there are some sorts of reports on the chemical properties of honey produced in the Tigray Region, there is a limitation of a scientific research on the quality of honeys produced in the Amhara Region making difficult to judge on the possible reason for the unbalanced preference. Therefore the objective of this study was to investigate physiochemical parameters and antioxidant and antimicrobial activities of honey samples collected from the two regions.

## 2. Materials and Methods

### 2.1. Description of the Study Areas

**Figure 1:**
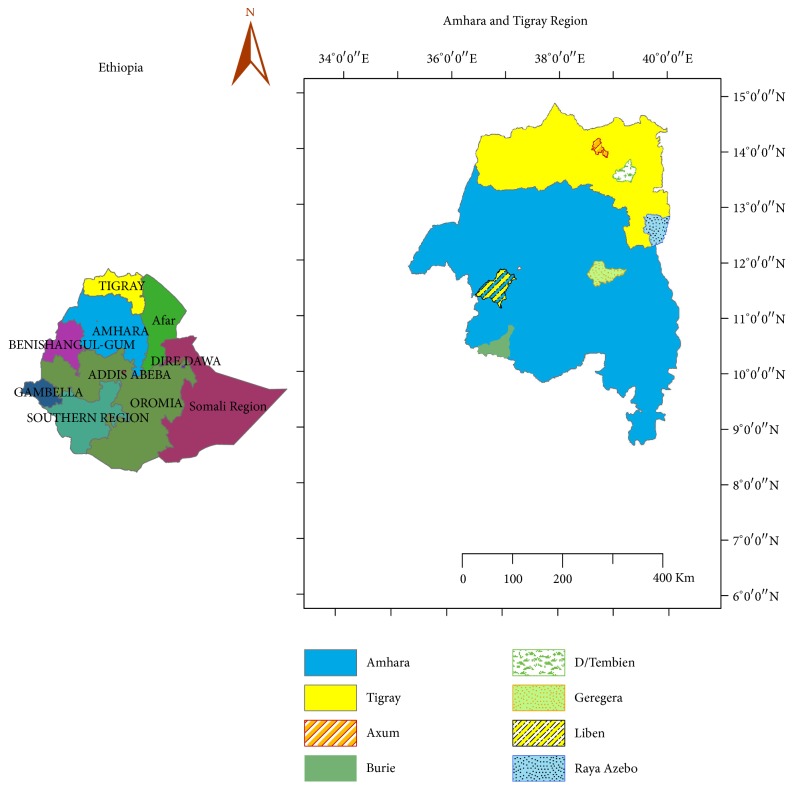
Location map of the study areas.

The study was conducted in six selected districts (Figure 1): three (Raya-Azebo, Degua-Tembien, and Axum) from Tigray Region and the other three (Geregera, Liben, and Burie) from Amhara Region.

### 2.2. Sample Size and Sampling Techniques

Three composite honey samples, each of 0.5 kg, were purchased directly from the farm gates of each district. A total of 18 composite honey samples were collected from the selected six districts of the two regions in October and November (harvesting season). All samples were collected in fresh, clean polyethylene bottle containers, labeled with numbers, place, and date of collection. The samples were then stored at room temperature until they were analyzed. Each composite honey sample was analyzed separately for the selected physicochemical parameters and antioxidant and antibacterial activities. After running a triplicate measurement for each parameter, a pooled mean of the three composite honey samples of each district was calculated.

### 2.3. Equipment and Reagents

UV-vis spectrophotometer (Agilent Technologies Carry 60 single beam UV-vis spectrophotometer), autoclave (portable pressure steam sterilizer with vessel volume of 0.018 m^3^ and working pressure of 0.14-0.16 MPa), electrical furnace (Thermo Scientific Thermolyne Muffle Furnace, USA), refractometer (Abbe pocket refractometer), water bath (serological thermostat, AI-7781, Germany), and pH meter (Hanna Instruments, Switzerland) were the equipment used.

Orthophosphoric acid (85%, Chem-Supply Pty Ltd., Australia), copper sulfate pentahydrate (99%, Jiangsu kolod Food Ingredients Co., Ltd.), sodium potassium tartrate tetrahydrate (Crystalline/Certified ACS, Fisher Chemical), sodium tungstate dihydrate and phosphomolybdic acid (Scharlau Chememia, USA), ethanol and methanol (Alpha Chemika, India), gallic acid (98%, Hefei TNJ Chemical Industry Co. Ltd., China), 2,2-diphenyl-1-picrylhydrazyl radical (DPPH), ascorbic acid (Sigma-Aldrich, USA), lead acetate (microtronics, QUALI-TECH, Amazon industrial and scientific store), and potassium oxalate (microtronics, QUALI-TECH, Amazon industrial and scientific store) were among analytical grade chemicals used in the research. Fehling's solution A, Fehling's solution B, Folin-Ciocalteu reagent, Muller Hinton Agar (MHA), nutrient broth, and 0.5 Mcfarland standard were prepared in the laboratory. Distilled water was used throughout the entire research work.

### 2.4. Methods

#### 2.4.1. Determination of Physicochemical Parameters of Honey

In this research, selected physicochemical parameters including moisture content, ash (mineral) content, EC, pH, free acidity, reducing sugar, sucrose content, and total phenolic content were determined.

*(1) Moisture Content.* The moisture content of honey, which is a measure of its stability and resistance to fermentation, was indirectly determined via measuring the refractive index of the sample at 20°C which is related to the water content according to the Wedmore table [13].

The digital refractometer was regularly calibrated at 20°C with distilled water so that the borderline between the white and dark area passed through the cross point of both lines visible in the ocular. The homogenized honey sample was evenly covered on the surface of the prism and the prism was closed for 4 minutes to stabilize. The water content of the samples was measured in triplicate and the average value was reported.

*(2) Ash (Mineral) Content.* The ash content of the honey samples which is believed to indicate that botanical origin was determined following the procedure by QSAE [14]. An ash dish was heated in an electric furnace at 600°C and subsequently cooled in a desiccator to room temperature and the dish was weighed (m_2_). Five grams of honey sample was weighed to the nearest 0.0001 g (M_0_) and added into the dish. Then water and other volatile components were removed by preliminary ashing using a hot plate.

After the preliminary ashing, the sample was ashed using a muffle furnace at 600°C for 6 hrs. The dish with the ash was then cooled in a desiccator for 30 minutes and the weight was recorded (m_1_). Then ash (% by mass) was calculated using the following formula:

(1) $$\begin{array}{c} Ash\;\;\left(\;\%\;\;by\;\;mass\;\right)=\frac{\left(m_{1}-m_{2}\right)}{M_{o}}*100 \end{array}$$

where m_2_ = weight of empty crucible, m_1_= weight of the ash and crucible, and Mo = mass of the sample taken for the test. 

*(3) Electrical Conductivity (EC).* Electrical conductivity of honey which depends on the ash, organic acids, proteins, some complex sugars, and polyols content [2] is a parameter that shows great variability according to the floral origin and is considered as one of the best parameters for differentiating between honeys with different floral origins [15].

In this research, 20 g of honey sample was dissolved in 100 mL distilled water. The electrical conductance of the specified solution was measured using the calibrated digital conductivity meter.

*(4) pH and Free Acidity.* Free acidity of honey refers to the total free acids expressed in meq /kg of honey [16]. These parameters have great importance during the extraction and storage of honey, as they influence the texture, stability, and shelf life [17]. Ten g of honey sample was dissolved in 75 mL of distilled water in a 250 mL beaker with the help of magnetic stirrer [18]. The pH of the solution was measured using a calibrated pH meter. To determine the free acidity, three drops of phenolphthalein were added as an indicator and the solution was titrated with 0.10 M NaOH (pH = 8.30). The free acidity of the samples was then calculated using the formula:

(2) $$\begin{array}{c} Free\;\;acidity\;\;\left(\;meq/kg\;\right)=10*V \end{array}$$

where V is the volume of NaOH consumed (mL). 

*(5) Reducing Sugar Content.* To determine the reducing sugar content of the studied honey samples, the modified method of the Lane and Eynon [19] was used. It involves the reduction of Fehling solutions by titrating against a solution of reducing sugars, while the solution is boiling at 60°C, using methylene blue as an internal indicator.

Two g (W) of the homogenized honey sample was dissolved in distilled water in a 100 mL volumetric flask. Two mL of 20% lead acetate solution was added to remove nonreducing interferents, shaken, and let to stand for 10 minutes. 10% potassium oxalate was added in small amounts until the precipitate was settled. This honey solution was filtered through Whatman No. 1 filter paper, diluted to the mark, and mixed well (honey solution). Fifty mL of the honey solution was diluted to 100 mL and used for titration.

To a 250 mL Erlenmeyer flask containing a mixture of 5 mL each of Fehling's solution A and Fehling's solution B, 8 mL distilled water was added followed by 15 mL diluted honey solution. The mixture was heated to boil over a hot plate at 60°C for 2 minutes. One mL of 0.2% methylene blue solution was added into the mixture while still boiling. The titration was done using the diluted honey solution until the indicator became decolorized. The result was calculated using the formula reported by Pearson [20]:

(3) $$\begin{array}{c} C=\frac{2}{W}*\frac{\text{1,000}}{V} \end{array}$$

where C = gram of invert sugar per 100 gram honey, W = weight (g) of honey sample, and V = volume (mL) of diluted honey solution consumed.

*(6) Sucrose Content.* The sucrose content of the honey samples was determined following reported procedure [20]. Honey solution was prepared as for determination of reducing sugar. Fifty mL of the honey solution was placed in a 100 mL volumetric flask that contained 25 mL distilled water and the mixture was heated to 65°C over a boiling water bath for an hour. The flask was then removed from the water bath and 10 mL of 6.34 M hydrochloric acid solution was added to it. The solution was allowed to cool for 15 minutes and neutralized with 10 mL of 5 M sodium hydroxide. It then cooled to 20°C and the volume was adjusted to 100 mL (diluted honey solution). Titration was done following similar procedure as for the determination of reducing sugar. The sucrose content expressed as gram of apparent sucrose per 100 g honey was calculated as

(4) sucrose  content=invert  sugar  content  after  inversion−invert  sugar  content  before  inversion∗0.95

*(7) Estimation of Total Phenolic Content.* The phenolic content of honey samples was estimated using a modified spectrophotometric Folin-Ciocalteu method [21]. 2.5 g of honey sample was diluted to 50 mL with distilled water and properly shaken and filtered through Whatman No. 1 filter paper. One mL of honey extract was mixed with 1 mL of Folin and Ciocalteu's phenol reagent. After 3 min, 1 mL of 10 % Na_2_CO_3_ solution was added to the mixture and diluted to 10 mL with distilled water. Finally, 3 mL of the mixture solution was added into the cuvettes and kept in the dark for 60 min, after which the absorbance was read at 760 nm. Gallic acid was used as a standard and the results were reported as mg of Gallic acid equivalents (GAEs) per kg of honey.

#### 2.4.2. Determination of Antioxidant Activity of Honey

Although many methods including DPPH, FRAP, ORAC, and TEAC are reported for determination of the antioxidant activity, the DPPH method, which is quick and simple test guarantees reliable results and only needs a UV-vis spectrophotometer [22], was used in this study.

Two g of honey sample was dissolved in 20 mL methanol at a concentration of 100 mg/mL, vigorously shaken, and filtered using filter paper. 0.5 mL of honey extract was mixed with 2.7 mL of methanolic solution containing DPPH radicals (0.024 mg/mL). The mixture was left in the dark for 15 min and the absorbance was measured at 517 nm. The natural antioxidant, ascorbic acid was used as a positive control [23]. The radical-scavenging activity (RSA) was calculated as the percentage of DPPH discoloration using the following:

(5) DPPH  scavengingactivity%=Acontrol−Asample∗100Acontrol

where A_control_ is the absorbance of the control and A_sample_ is absorbance of the sample solution.

IC_50_, which is the concentration of honey sample required to scavenge 50% of DPPH^•^, was determined using the plot of RSA (%) as a function of different honey concentrations [24].

#### 2.4.3. Determination of Antibacterial Activity of Honey

Undiluted honey samples were screened for their antimicrobial activity by the agar well diffusion method [25]. This antimicrobial activity test was conducted using one gram positive (*Staphylococcus aureus (*ATCC 25923) and two gram negative (*Salmonella typhi* (ATCC6539 and* Escherichia coli (*ATCC25922)) bacteria [4]. Bacterial strains of the test organisms were grown overnight in the nutrient broth prepared by dissolving 8 gram nutrient broth powder in one liter of distilled water. The mixture was mixed thoroughly to form a clear medium which was incubated at 37°C for 24 hours after the bacterial specimens were inoculated. The small colonies of the test organisms were made into suspension with 1 mL of sterile distilled water in the test tubes followed by the addition of 5 mL of a density of a 0.5 McFarland standard (a standard which was used to prepare bacterial suspensions to a specified turbidity). In the Kirby-Bauer disk diffusion susceptibility test protocol, the bacterial suspension of the organism to be tested should be equivalent to a 0.5 McFarland standard. To do this adjustment, the tubes containing the bacterial suspension and the McFarland standard were held side by side in order to see the appearance of the lines through both suspensions. The adjustment process was completed by the additions of more organisms/ saline, based on the appearance, to the inoculums tube. 0.1 mL of each bacterial suspension, equivalent to 0.5 McFarland's standard, was evenly distributed into sterilized petridishes that contained 15 mL of melted and sterilized Muller Hinton nutrient agar maintained at 45°C.

The inoculation of the bacteria was done by streaking the surface of the plates with a sterile swab until the entire surface was covered. The plates were allowed to set; three equidistant wells of 4 mm in diameter were punched in each plate using a sterile cork borer. To each of the wells, 0.2 mL of pure undiluted honey, heated to 30°C for 30 minutes, filtered, and incubated overnight, was introduced. A well filled with sterile water was served as control and the plates were allowed to stay for 15 minutes for prediffusion to take place followed by incubation for 48 hrs at 37°C. The inhibition zones were measured using a metric rule.

### 2.5. Statistical Analysis

The results of all experiments were expressed as mean ± SD of triplicate measurements. Honey quality data were analyzed by MS Excel 2007. Origin software was used to draw the calibration curves of the standards. One-way ANOVA using SPSS software version 22 followed by Tukey's post hoc multiple comparisons test was used to check the statistical differences among quality indicator parameters of the honey samples from different districts. Values with P < 0.05 were considered statistically significant.

## 3. Results and Discussion

### 3.1. Physicochemical Parameters

#### 3.1.1. Moisture Content

The average moisture content of studied honey samples was in the range 16.34 ± 0.26 to 19.83 ± 0.43% with a mean value of 18.09 ± 1.23 (Table 1). These values were under the recommended values of the National (21%), International (18-23%) and WHO/FAO (21-23%) standards.

According to the national classification of honeys based on their moisture content [14], honey samples from Geregera and R/Azebo belonged to grade A (17.5-19.0%) while samples from Burie and Liben belonging to grade B (19.1-20.0%).

Moreover, honey samples from Axum, D/Tembien, R/Azebo and Geregera could be placed in grade A (16-18.6%) of United States standard [26]. The ANOVA result revealed statistically significant difference (P < 0.05) of moisture content among the honey samples collected from different districts. However, the post hoc multiple comparisons test indicated that there was no significant difference (P > 0.05) between the honey samples from Geregera and D/Tembien. The variations may be due to the difference in the climatic conditions of the environment and/or the degree of maturation the honey reached in the hive.

The moisture contents of honey samples collected from the districts of Tigray Region in general were lower as compared to the honey samples from the districts of Amhara Region which might be attributed to the prevailing atmospheric humidity both before and after removal of honey from the hives, as the climatic condition of Tigray is characterized by low humidity of air.

In spite of slight numerical variations, the moisture content results of this study were in agreement with the literature values reported by Kebede et al. [3] ( 18.6–18.8%), USDA [27] (16-18.6%) for grade “A” and (18.7-20%) for grade “B” honeys, Tewodros et al. [28] (13.9-17.7%), and Gebru et al. [29] (17.5–23%).

#### 3.1.2. Ash (Mineral) Content

The mean ash content of the studied honey samples varied from 0.08 ± 0.00 to 0.45 ± 0.03% with a mean value of 0.21 ± 0.01 (Table 1). The mineral contents of all honey samples were within the acceptable range of the national (< 0.6) and international (0.02-1) standards of honey quality. The ANOVA test indicated statistically significant difference (P < 0.05) of ash content among the honey samples from different districts. This study also indicated that honey samples collected from the Amhara sampling districts were found to have higher mineral content as compared to the honey samples from Tigray which could be due to the difference in botanical origin, materials gathered by the bees, soil composition, and environmental conditions. Due to the fact that honeys collected from the Tigray Region were more white than honeys from the Amhara Region, higher ash content of the samples from the Amhara districts than the samples from Tigray could also be ascribed to the sample color difference [29].The results of this study were in agreement with the literature values reported by Tewodros et al. [28] (0.0-0.52%), Gebru et al. [29] (0.09-0.3%), and Adisu and Malede [30]) (0.014–0.31%).

#### 3.1.3. Electrical Conductivity

The electrical conductivity results of all studied honey samples ranging from 0.19 ± 0.00 to 0.89 ± 0.03 mS/cm with a mean value of 0.40 ± 0.02 mS/cm (Table 1) were found within the acceptable range (0.1-3 mS/cm) of the International standard [9].

According to the international norm specified by both Codex Alimentarius Commission and European Council (EU) [1], the honey sample from Burie with the electrical conductivity of 0.89 ± 0.03 mS/cm may be considered as honeydew honey (≥0.8 mS/cm) while honey samples from the other districts may be categorized as multifloral blossom honeys (<0.8 mS/cm). The highest electrical conductivity value of the honey from Burie district was in agreement with its highest mineral content (0.45 ± 0.03 mS/cm).

The ANOVA test showed statistically significant difference (P < 0.05) among the analysed honey samples, although the post hoc multiple comparison test showed there was no statistically significant difference (P > 0.05) between the honey samples from D/Tembien and R/Azebo. The observed significant variations in electrical conductivity may be due to the possible variation in degree of maturity, the soil composition, and/or the environmental conditions.

**Figure 2:**
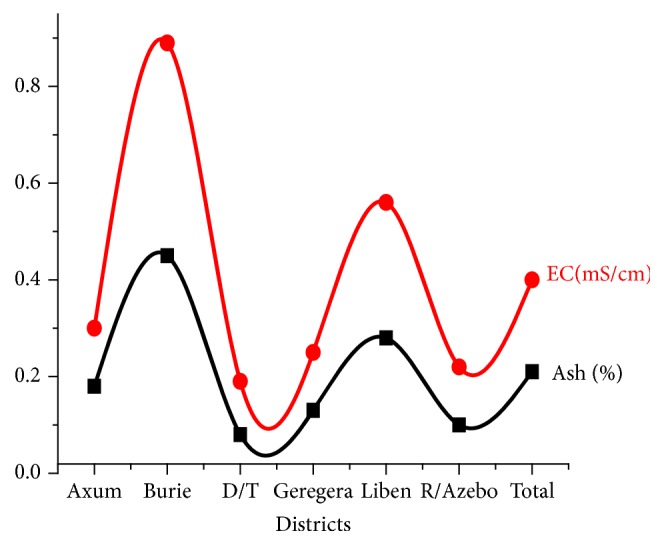
Relationship between ash content and EC values of the studied honey samples.

The electrical conductivity of all the studied honey samples showed similar trends to the mineral contents (Figure 2) and was in line with literature values reported by Gebru et al. [29]) (0.25–0.41 mS/cm) and Adisu and Malede [30] (0.41-72 mS/cm).

#### 3.1.4. pH and Free Acidity

The pH of the studied honey samples varied from 3.79 ± 0.04 to 4.20 ± 0.01 with a mean value of 3.95 (Table 1), while the conventional permissible pH value of honey is in the range 3.2 to 4.5 [31], of blossom honeys is in the range 3.2 and 4.6 [32], and of honeydew honeys is in the range 4.5 and 6.5 [32]; the studied honeys are therefore satisfying the conventional standard [31] and the blossom honey range [32]. The post hoc multiple comparison test revealed no significant difference (P > 0.05) in pH between honey samples from Geregera and Liben although the ANOVA analysis result indicated significant difference (P < 0.05) among the studied honey samples. Though there are slight numerical variations, the results of this study were in line with literature values reported by Tewodros et al. [28] (3.55–4.75), Gebru et al. [29] (3.89–4.450), and Adisu and Malede [30] (4.16-4.27).

The free acidity of the studied honey samples expressed in meq/kg ranged from 19.56 ± 1.13 to 38.11 ± 1.54 with a mean value of 29.74 ± 2.20 was all within the recognized national (< 40 meq/kg) [14], international [9] (5-54 meq/kg), and WHO/FAO (< 40 meq/kg) [4] standards of honey quality. The results of this study were comparable with literature values reported by Gebru et al. [29] (17.33-32.7 meq/kg) and Adisu and Malede [30] (18.01-38.8 meq/kg) indicating that the analyzed honey samples were good in their taste, free from unwanted fermentation, and capable of inhibiting microbial growth in this range of acidity.

#### 3.1.5. Reducing Sugar Content

The reducing sugar contents of the studied honey samples were found in the range of 62.10 ± 0.48 to 66.37 ± 0.24% with a mean value of 64.93 ± 1.53% (Table 1). The honey samples from Geregera, Axum, and D/Tembien exhibited a reducing sugar content of above 65% and hence were in agreement with the standards set by QSAE [33] and Codex [34]. As can be seen from the table, the reducing sugar contents of the rest honey samples were found to be slightly lower than the above standards but they were still in agreement with the international standard (60–70%) of honey quality.

While the ANOVA test showed a significant difference (P < 0.05) in reducing sugar content among the studied honey samples the post hoc multiple comparisons test revealed no significant difference (P > 0.05) among the honey samples from Geregera, Axum, and D/Tembien. The variations might be due to the variation of the plant sources from which the honeys were produced and/or the degree of maturation the honey reached in the hive. The results of this study were in agreement with the literature values for Ethiopian honeys reported by Tewodros et al. [28] (63.4–71.7%), Adgaba [35] (mean of 65.5%), and Bekele et al. [36] (51.28–69.2%).

#### 3.1.6. Sucrose Content

The sucrose content of the analyzed honey samples ranged from 1.35 ± 0.08 to 5.96 ± 0.10% with a mean value of 3.6 ± 0.28% (Table 1), while the sucrose content of all studied honey samples was below the international limit (<10%); the honey samples also showed sucrose content within the national limit (<5%) except that the honey from Axum revealed that it was slightly higher than the national standard value. The low sucrose content of the studied honey samples indicated that honeys produced from the study areas were natural and free of any adulteration [37].

The statistical analysis of variance revealed significant difference (P < 0.05) among the honey samples from different districts whereas the post hoc multiple comparison test showed no significant difference (P > 0.05) between the honey samples from Geregera and D/Tembien. The observed sucrose's content significant difference might be due to the variation of the geographical locations the plants on which the bees forage and the degree of maturation [37].

Despite slight numerical variations, the sucrose content results of this study were in agreement with literature values reported by Tewodros et al. [28] (1-5.2%) and Eyobel et al. [38] (2.28-5.72%).

#### 3.1.7. Estimation of Total Phenolic Content

**Figure 3:**
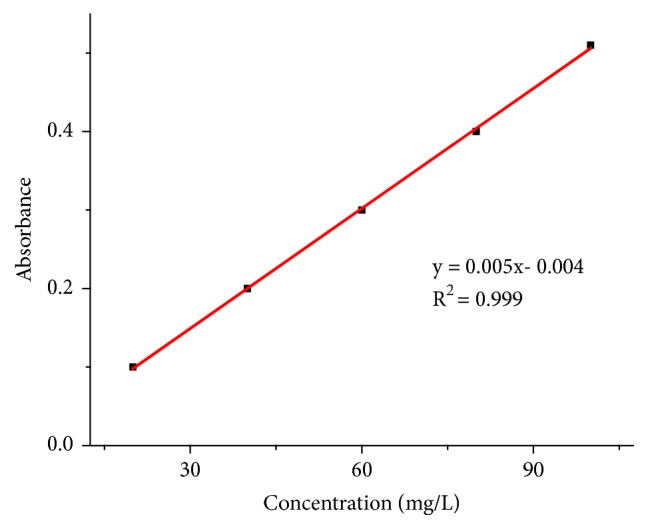
Plot of absorbance of FCR in the presence of various concentrations of gallic acid.

The total phenolic content of the current honey samples was estimated using five series of working standard solutions of gallic acid (0.01, 0.02, 0.04, 0.06, 0.08, and 0.10 mg/L), and the resulting* absorbance versus concentration* calibration curve is shown in Figure 3.

The total phenol contents of the studied honey samples were found in the range of 1165.60 ± 23.45 to 1854.83 ± 10.47 mg/kg (Table 2). Although statistical analysis results indicated significant difference (P < 0.05) in the total phenolic content among the studied honey samples, honey samples from the districts of Amhara Region were generally found to have higher phenolic content than honey samples from Tigray. The variations might be due to the difference in the geographical origin of the plants from which honeys were produced and/or the color variation of the honey samples [39]. The results of this study were somewhat in agreement with reported literature values (65.31 mg GAE/100 g) [40] on the total phenolic content and antioxidant activities of natural honeys and propolis from different geographical Regions of Ethiopia.

### 3.2. Antioxidant Activity of Honeys

**Figure 4:**
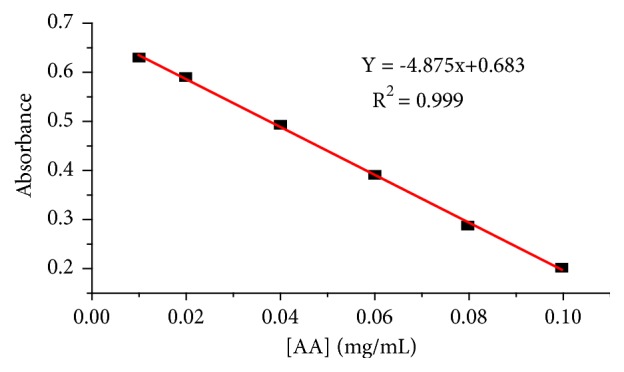
Plot of absorbance of DPPH as a function of various concentrations of ascorbic acid.

The antioxidant activity of the tested honey samples was estimated using six series of working standard solutions of ascorbic acid, and the resulting absorbance* versus *concentration calibration curve is presented in Figure 4.

Due to the neutralizations of the DPPH free radical by ascorbic acid, the calibration curve moves downwards with an increment of ascorbic acid concentration. Before reacting with ascorbic acid, DPPH solution was purple in color and had a maximum absorbance; however, when it was mixed with ascorbic acid solution and allowed to stand for 15 minutes in the dark place, the purple color of the solution was turned to yellow (Owen laboratory observation). The calculated antioxidant activities of the studied honey samples were in the range of 21.64 ± 0.26 to 36.12 ± 0.52 mg AEAC/100 g honey (Table 2) which were in line with their total phenolic content. This confirmed that phenolic compounds are the responsible components for the antioxidant activity of honeys. But this does not mean that honeys with the highest phenolic content will always have the highest antioxidant activity [41].

Analysis of variance showed statistically significant difference (P < 0.05) in antioxidant activities among the honey samples which might be attributed to the variation of the geographical locations and the botanical origins of the plants from which the honeys were produced and hence chemical composition [39].

The antioxidant activity results of the studied honey samples followed the reported trend [40, 42] showing darker honeys have higher antioxidant activity than white honeys.

Another antioxidant parameter investigated in this study was the IC_50_ value of the honey samples. It is the amount of antioxidant required to scavenge 50 % of the free radical.

**Figure 5:**
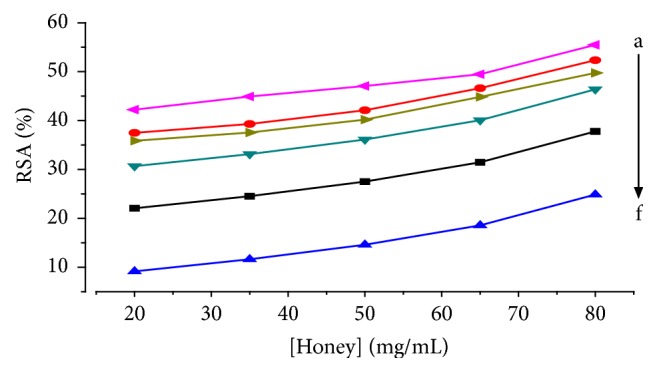
DPPH RSA of the honey samples (a-f: Liben, Burie, R/Azebo, Geregera, Axum, and D/Tembien, respectively) at various concentrations.

As indicated in Figure 5, honey sample from Liben showed the highest RSA and honey sample from D/Tembien showed the lowest RSA in all concentration ranges. The highest RSA was recorded at the highest honey concentration (80 mg/mL) while the lowest RSA was obtained at the lowest honey concentration (20 mg/mL).

The IC_50_ values of the investigated honey samples ranged from 60.48 to 184.45 mg/mL (Table 3). Ascorbic acid, which was used as a positive control, was found to have the IC_50_ value of 0.021 mg/mL with the RSA of 82.79 %, indicating its highest antioxidant activity than the tested honey samples.

The honey samples in this study showed lower IC_50_ values than reported literature values [43] indicating that honey samples of the present study were more efficient in their antioxidant activity than heterofloral Croatian honeys. Moreover, the results were in line with previous results for Ethiopia honeys [40].

Analysis of the correlation between total phenolic content and antioxidant activity of the studied honey samples revealed (Table 4) that the total phenolic contents of the honey samples were strongly correlated with their antioxidant activities with the correlation coefficient (r) of 0.94.

### 3.3. Antibacterial Activity of Honeys

The antibacterial activity of the tested honey samples against* Salmonella typhi* varied from 23.23 ± 0.12 to 28.84 ± 0 .24 mm (Table 5). Similarly, the inhibition zones ranged from 23.47 ± 0.09 to 27.26 ± 0.09 mm and 23.39 ± 0.06 to 25.38 ± 0.08 mm for* Escherichia coli* and* Staphylococcus aureus*, respectively. In the study, honey sample from Axum induced the highest inhibition zones against all the three tested bacterial species and honey sample from Liben exerted the lowest inhibition zones.

The ANOVA test showed statistically significant difference (P < 0.05) in inhibition zones among the studied honey samples (Table 5). The variations may be due to the difference in the sugar content, moisture content, and acidity [44, 45].

In accordance with the Baur et al. [46] antibacterial drug classification is based on bacterial sensitivity as “resistant” if an induced zone of inhibition by an antimicrobial drug is less than 8 mm, “intermediate” if it is between 8 and 11 mm, and “susceptible”, if it exerted an inhibition zone diameter of 12 mm or more; all tested bacterial strains in this study could be considered as “susceptible” to the antibacterial effect of the tested honey samples.

Although the antibacterial activity results of this study were in agreement with most reported results [47, 48], they were somewhat higher than the values reported by Mohammed et al. [49]

## 4. Conclusion

The study was conducted to assess selected physicochemical parameters and antioxidant and antibacterial activities of honey samples from selected districts of the Amhara and Tigray Regions. The moisture content, electrical conductivity, mineral content, and pH and free acidity values of all honey samples were found to be within the acceptable limit of the national and international standards. Although the honey samples from the districts of Tigray Region showed higher reducing sugar content than the honey samples from the districts of Amhara Region, they all were within the international standard and no substantial mean differences were found.

Honey samples from the districts of Amhara Region exhibited higher total phenolic content and better antioxidant activity than the honey samples from Tigray. The total phenolic contents of honey samples were strongly correlated with the antioxidant activity. Furthermore, all honey samples showed antibacterial activity. Significant differences (P < 0.05) were found in the investigated parameters among the honey samples from different districts. Therefore, most of the results in this study do not support the peoples' preference for white honey from Tigray over white honey from Amhara Region.

## Figures and Tables

**Table 1:** Physicochemical composition of honey samples of the study areas.

Districts	MC (%)	Ash (%)	EC(mS/cm)	pH	Free acidity (meq/kg)	Reducing sugar (%)	Sucrose (%)
Axum	16.34 ± 0.26^e^	0.18± 0.01^c^	0.30 ± 0.01^c^	3.85 ± 0.01^d^	34.11 ± 1.05^b^	66.37 ± 0.24^a^	5.96 ± 0.10^a^
D/Tembien	17.34 ± 0.22^d^	0.08 ± 0.00^f^	0.19 ± 0.00^e^	4.00 ± 0.08^b^	24.67 ± 1.14^e^	66.03 ± 0.56^a^	2.65 ± 0.16^d^
R/Azebo	18.44 ± 0.54^c^	0.10± 0.00^e^	0.22 ± 0.01^e^	3.79 ± 0.04^e^	38.11± 1.54^a^	64.30 ± 0.78^c^	4.24 ± 0.06^c^
Burie	19.09 ± 0.43^b^	0.45 ± 0.03^a^	0.89 ± 0.03^a^	4.20 ± 0.01^a^	19.56 ± 1.13^f^	64.93 ± 0.38^b^	1.35 ± 0.08^e^
Geregera	17.52 ± 0.43^d^	0.13 ± 0.01^d^	0.25 ± 0.01^d^	3.91 ± 0.01^c^	29.67 ± 1.00^d^	65.89 ± 0.20^a^	2.69 ± 0.13^d^
Liben	19.83 ± 0.43^a^	0.28 ± 0.02^b^	0.56 ± 0.05^b^	3.89 ± 0.05^c^	32.33 ± 2.65^c^	62.10 ± 0.48^d^	4.86 ± 0.31^b^
Overall mean	18.09 ± 1.23	0.21 ± 0.01	0.400 ± 0.02	3.94 ± 0.14	29.74 ± 2.20	64.93 ± 1.53	3.6 ± 0.28
World Std.	18-23	0.02-1	0.1-3	3.4-4.5	5–54	60-70	< 10

Values are presented as mean ± SD of three determinations. Values with different superscripts down the column are significantly different (P < 0.05).

**Table 2:** Total phenolic content and antioxidant activity of the studied honey samples.

Districts		TPC(mg/kg honey)	AEAC/100 g honey	% RSA
Tigray	Axum	1393.06 **±**11.64^e^	24.02 **± **0.34^e^	51.28 **± **0.15^e^
	D/Tembien	1165.60 **± **23.45^f^	21.64 ± 0.26^f^	50.24 **± **0.13^f^
	R/Azebo	1565.27 **± **7.70^d^	29.37 ± 0.26^c^	53.55 **± **0.12^c^
Amhara	Burie	1747.51 **± **17.26^b^	34.80 ± 0.37^b^	55.78 **± **0.16^b^
	Geregera	1595.80 **± **7.79^c^	26.20 ± 0.31^d^	52.22 **± **0.15^d^
	Liben	1854.83 **± **10.47^a^	36.12 ± 0.52^a^	56.36 **± **0.22^a^

TPC = total phenolic content, AEAC = ascorbic acid equivalent antioxidant capacity, RSA = radical scavenging activity, and IC_50_ = 50 % inhibitory concentration. Values with different superscripts down the column are significantly different (P < 0.05).

**Table 3:** Antioxidant activity of honey samples in terms of their IC_50_ values.

Districts		Regression Equation	R^2^	IC_50_ (mg/mL)
Tigray	Axum	*y* = 0.255*x* + 15.870	0.962	133.84^b^
	D/Tembien	*y* = 0.255*x* + 2.966	0.962	184.45^a^
	R/Azebo	*y* = 0.233*x* + 29.950	0.959	86.05^d^
Amhara	Burie	*y* = 0.246*x* + 31.230	0.954	76.30^e^
	Geregera	*y* = 0.255*x* + 24.480	0.962	100.08^c^
	Liben	*y* = 0.207*x* + 37.480	0.949	60.48^f^
*Ascorbic acid *		*y* = 419.5*x* + 41.170	0.999	0.021

Values with different superscripts down the column are significantly different (P < 0.05).

**Table 4:** Correlations between total phenolic contents and RSA of honey samples.

		TPC	AEAC	RSA	IC_50_
TPC	r	1			
AEAC	r	0.938*∗∗*	1		
RSA	r	0.940*∗∗*	1.000*∗∗*	1	
IC50	r	- 0.884*∗∗*	- 0.968*∗∗*	- 0.970*∗∗*	1

*∗∗* correlation is significant at 0.01 level.

**Table 5:** Antibacterial potential of the tested honey samples against selected bacterial species.

Districts		Tested Bacteria with Inhibition Zones		
		*S.typhi* (mm)	*E.coli* (mm)	*S.aureus* (mm)
Tigray	Axum	28.84 ± 0 .24^a^	27.26 ± 0.09^a^	25.38 ± 0.08^a^
	D/Tembien	25.59 ± 0.12^b^	25.28 ± 0.11^b^	25.01 ± 0.28^b^
	R/Azebo	23.68 ± 0.12^e^	23.90 ± 0.20^e^	23.64 ± 0.11^d^
Amhara	Burie	24.17 ± 0.10^d^	24.14 ± 0.10^d^	23.77 ± 0.09^d^
	Geregera	24.60 ± 0.24^c^	24.67 ± 0.46^c^	24.22 ± 0.08^c^
	Liben	23.23 ± 0.12^f^	23.47 ± 0.09^f^	23.39 ± 0.06^e^

Values are presented as mean ± SD of three determinations. Values with different superscripts down the column are significantly different (P < 0.05).

## Data Availability

The data used to support the findings of this study are available from the corresponding author upon request.
